# Mebendazole Shows Antiproliferative and Antimigratory Effects in Paediatric Low-Grade Glioma Models

**DOI:** 10.32604/or.2026.071074

**Published:** 2026-06-16

**Authors:** Chiara Ferraro, Michela Pizzoferrato, Michela Graziano, Tiziana Servidei, Antonio Ruggiero, Pierluigi Navarra, Lucia Lisi

**Affiliations:** 1 Section of Pharmacology, Department of Translational Medicine and Surgery, Catholic University Medical School, Fondazione Policlinico Universitario A. Gemelli-IRCCS, Rome, Italy; 2 Pediatric Oncology Unit, Catholic University Medical School, Fondazione Policlinico Universitario A. Gemelli-IRCCS, Rome, Italy

**Keywords:** Pediatric low-grade glioma, drug repositioning, Mebendazole, Mitogen-Activated Protein kinase signaling

## Abstract

**Objectives**: Advances in molecular profiling of pediatric low-grade glioma have enabled targeted therapies to emerge as effective and better-tolerated alternatives to conventional chemotherapy, increasingly used in progressive or recurrent disease and may reduce long-term treatment toxicity. This study aimed to evaluate the repositioning of the anthelmintic drug mebendazole (MBZ) as an antiproliferative agent in pediatric glioma models, and to investigate potential synergistic effects in combination with vinblastine. **Methods**: Two well-established human pediatric glioma cell lines, RES 259 and RES 186, were exposed to MBZ alone or in combination with vinblastine. Cell viability, cytotoxicity, and tumor invasiveness were assessed using functional assays. The effects of MBZ on intracellular signaling pathways, particularly Mitogen-Activated Protein (MAP) kinase and on migration-related proteins, were further analyzed. **Results**: MBZ treatment for 48 h induced cytotoxicity and significantly inhibited cell growth in both RES 259 and RES 186. In addition, MBZ demonstrated anti-migratory and disrupted MAP kinase signaling. Experiments investigating combination treatment revealed that MBZ and vinblastine did not exert synergistic or additive effects, likely due to their shared targeting of microtubule dynamics. **Conclusion**: These findings indicate that MBZ exerts potent antiproliferative and anti-migratory activity in pediatric glioma cell lines, supporting its potential as a repositioned therapeutic drug. However, no additional benefit was observed when combined with vinblastine, underscoring the importance of exploring MBZ as a single-agent strategy in future translational studies.

## 1 Introduction

Tumors of the central nervous system (CNS) are the most frequent solid tumors in children, with approximately 5.4–5.6 diagnoses per 100,000 [1,2]. Comprising over 30% of pediatric CNS neoplasms, low-grade gliomas (LGG) and glioneuronal tumors are the most prevalent brain tumors in children, despite their low overall incidence in the general population [3,4,5,6].

Management of Pediatric low-grade glioma. In pediatric low-grade glioma (pLGG), the extent of surgical removal is a key determinant of clinical outcome, with maximal resection consistently associated with improved prognosis [2]. Tumors located near the brain surface—such as those in the cerebral hemispheres or posterior fossa—are often amenable to complete excision. Conversely, deeply seated or highly infiltrative lesions frequently limit the feasibility of radical surgery [7]. When residual tumor persists or disease progression occurs, patients have traditionally been managed with postoperative chemotherapy or radiotherapy [8,9,10]. Vincristine and carboplatin combinations, or vinblastine monotherapy, remain the most frequently used chemotherapy regimens for the treatment of pLGG in many centers worldwide [11,12,13].

Molecular insights have fundamentally reshaped the understanding of pLGG biology, showing that dysregulation of the RAS/MAPK pathway is a common underlying mechanism across these tumors, often due to a mutation or fusion of the B-rapidly accelerated fibrosarcoma (BRAF) gene [14,15,16].

Based on this evidence, targeting hyperactivation of the RAS-MAPK pathway has shown promise in pediatric low-grade gliomas (pLGG), with RAF and MEK inhibitors—such as vemurafenib, dabrafenib, trametinib, and selumetinib—demonstrating efficacy in preclinical and clinical studies [17]. Advances in tumor sequencing and gene expression analyses are enabling precision oncology approaches, allowing identification of driver mutations, refined diagnoses, and improved therapy selection, as shown by the INFORM registry. Current trials, including LOGGIC/FIREFLY-2, are generating extensive tumor sequencing datasets, supporting the use of transcriptome-based signatures to predict drug sensitivity and guide patient stratification in a clinically relevant and personalized manner [18].

While pLGGs carry a favorable prognosis for survival, they often cause lasting physical and developmental harm. Managing these cases requires a delicate balance between stopping tumor growth and preventing toxicities that affect vision, hormones, and brain development Future treatments are therefore moving beyond simple survival, focusing instead on reducing long-term side effects and improving patients’ quality of life [11,12,13].

Mebendazole (MBZ), chemically known as methyl 5-benzoyl-1*H*-benzimidazol-2-yl-carbamate, is a broad-spectrum anthelmintic drug that is used to treat various parasitic worm infections in humans. FDA authorization allows the use of this agent in children and adults over two years of age to treat gastrointestinal disease associated with a broad range of parasites [19,20].

International recommendations from the World Health Organization (WHO) support prolonged Mebendazole therapy at weight-based dosing (40–50 mg/kg/day), with markedly different treatment courses for cystic versus alveolar echinococcosis [21]. Final dosing strategies are tailored to the underlying helminthic infection [22].

MBZ works by interfering with the parasites’ ability to absorb glucose, which is essential for their survival, ultimately leading to their death [23]. MBZ is known to block microtubule functions of parasites and mammalian cells. This interference occurs through the inhibition of α/β tubulin polymerization, achieved by binding to the colchicine binding-site of β-tubulin [20]. This, in turn, disrupts the formation of microtubules, leading to the inhibition of glucose uptake and transport, which eventually leads to a shortage of glycogen [24].

Some studies have reported its anticancer effects on various cancer types, both *in vitro* and *in vivo* [25,26,27]. A broad spectrum of anticancer actions has been described for benzimidazole compounds such as MBZ: these agents limit tumor cell growth and dissemination, alter microtubule dynamics, and trigger programmed cell death and autophagic pathways. They further affect tumor biology by reducing angiogenesis, metabolic activity, and multidrug resistance, while promoting differentiation, senescence, and multinucleation [28] By interfering with microtubules, MBZ disrupts the normal progression of the cell cycle in cancer cells. This disruption can lead to cell cycle arrest, preventing cancer cells from dividing and proliferating. By inhibiting angiogenesis, MBZ may help restrict the blood supply to tumors, thereby inhibiting their growth.

The potential of MBZ to inhibit cancer cell growth has been documented in a range of cancer types, including thyroid [29], gastrointestinal [30], breast [31], prostate [32], pancreatic [25], ovarian [33], colorectal [34], melanoma [35], head and neck [36], leukemia [37] and bile duct cancer [38].

MBZ exhibited IC_50_ values in the nanomolar to micromolar range across all cell lines [31]. In addition, the therapeutic appeal of MBZ lies in its selective cytotoxicity; while it remains largely inert toward healthy tissues, it exhibits a heightened affinity for triggering cell death within malignant populations [22].

MBZ has demonstrated its ability to inhibit various signaling pathways, including MAPK/Signal transducer and activator of transcription 3 (STAT3), c-Jun N-terminal kinase (JNK), ELK/Serum Response Factor (SFR), V-Myc Myelocytomatosis Viral Oncogene Homolog/MYC associated factor X (MYC/MAX) and Nuclear factor kappa-light-chain-enhancer of activated B (NF-κB), leading to apoptosis, autophagy and DNA damage in various cancer types [31,33,39]. Within the MAPK signaling pathway, benzimidazole anthelmintics inhibited Extracellular signal-regulated kinases (ERK) activation [40] but increased p38 and JNK activations [41].

Benzimidazole anthelmintics inhibited cell migration and invasion, associated with the epithelial-to-mesenchymal transition (EMT). These effects are manifested through decreased levels of N-cadherin, vimentin, focal adhesion kinase (FAK) and matrix metalloproteinase (MMP) 2. Inhibition of FAK and MMP-2 by benzimidazole anthelmintics disrupts the mesenchymal program, leading to decreased metastasis [28]. These agents further enhance autophagic signaling, with upregulation of proteins including LC3 and beclin-1 [42]. In endothelial cells, mebendazole-induced autophagy may interfere with angiogenesis, potentially through modulation of the wingless-type MMTV integration site family (Wnt)/β-catenin axis and Vascular Endothelial Growth Factor Receptor 2 (VEGFR-2) levels [43].

This study aimed to investigate the effects of MBZ on cell viability, toxicity and tumor invasiveness in well-established human paediatric glioma models [44]. Experiments aimed to investigate putative synergistic or additive effects with Vinblastine were also carried out.

## 2 Methods

### 2.1 Materials

MBZ (HY-17595, CAS No. 31431-39-7, Polymorph C) was purchased from MedChemExpress (Monmouth Junction, NJ, USA) and dissolved in dimethyl sulfoxide (DMSO) to a stock concentration of 10 mM. Serial dilutions were made in cell culture media prior to cell treatments.

### 2.2 Cell Cultures

Low-grade pediatric glioma cell lines, RES 186 (RRID: CVCL_DG03) and RES 259 (RRID: CVCL_DG10), were kindly provided by Professor Antonio Ruggiero. RES186 and RES 259 were derived from a 3-year-old female patient with pilocytic astrocytoma and a 4-year-old female patient with diffuse astrocytoma, respectively [44]. Mycoplasma contamination was excluded before cell freezing using the Venor^®^ GeM Classic kit (Minerva Biolabs; Cat. No.11-1025). Pediatric glioma cell lines were cultured in Ham’s F-12 Nutrient Mixture (Sigma-Aldrich, St. Louis, MO, USA; Cat. No. D6421) supplemented with 10% Fetal Bovine Serum (FBS) (Gibco, Thermo Fisher Scientific Inc., Waltham, MA, USA; Cat. No. 10091-148), 100 U/mL penicillin-streptomycin (Gibco, Thermo Fisher Scientific Inc., Waltham, MA, USA; Cat. No. 30-002-CI) and 2 mM L-glutamine (Corning, New York, NY, USA; Cat. No. G7513) and were maintained at 37°C and 5% CO_2_. Cells were grown to 80% confluency, then split and resuspended in an appropriate volume of fresh medium. All experiments were performed in a culture medium also containing 1% FBS. The experiments were performed at different times (48 h, 72 h and 7 days of treatment), depending on the objective of the experiment.

### 2.3 Metabolic Activity Assay

In order to assess Mebendazole’s effect on metabolic activity at different time points, XTT (TACS^®^ XTT Cell Proliferation Assay Kit, R&D Systems, Minneapolis, MN, USA; Cat. No. 4891-025-K) was performed, according to the manufacturer’s procedure. Metabolic activity was measured by measuring the reduction of formazan salts with absorbance at 490 nm, which is read with a plate spectrophotometer (Victor 4, Perkin Elmer Inc., Waltham, MA, USA). In these experiments, for both cell lines, 10,000 cells/well were plated for the 24–48 h and 6-day assays, in 96-well plates. After 24 h from plating, the culture medium was removed and cells were treated with different concentrations of Mebendazole (ranging from 1 nM to 100 μM), dissolved in medium containing 1% FBS. For the 6-day experiments, media containing test substances were replaced with fresh media containing test substances every 24 h. After 24 h–48 h or 6 days of incubation, 50 μL of the XTT reagent, provided in the kit mentioned above, was added for a further 2 h. Two absorbance readings were performed after the XTT reagent addition, one at time 0 and one after 2 h, in order to subtract nonspecific absorbance. Half-maximal inhibitory concentrations (IC_50_) were calculated by nonlinear regression using a four-parameter logistic (4PL) model applied to log_10_-transformed concentration–response data. The IC_50_ value was determined as the drug concentration corresponding to a 50% reduction in viability, as defined by the intersection between the fitted curve and the 50% viability reference line. In cases where 50% inhibition was not reached within the tested range, the IC_50_ was reported as greater than the highest concentration tested. Finally, the effect of vinblastine on the metabolic activity of both cell lines was also evaluated after a 48-h treatment. The drug was used at concentrations ranging from 1 pM to 100 nM.

### 2.4 Lactate Dehydrogenase (LDH) Assay

To assess the potential toxicity effect of Mebendazole on RES 186 and RES 259 cells, the LDH assay (CytoTox 96^®^ Non-Radioactive Cytotoxicity Assay, Promega, Madison, WI, USA, Cat. No. G1780) was performed, following the manufacturer’s instructions. Cell toxicity was evaluated by first measuring the activity of lactate dehydrogenase (LDH) released in the culture supernatant and subsequently in the cell lysate obtained from the same wells, in order to determine the total LDH content. Cells were plated at a density of 10.000 cells/well for the experiments conducted at 48–72 h. To quantify lactate dehydrogenase (LDH) levels, absorbance was recorded at 490 nm using a Victor 4 microplate reader, applying a 650 nm reference correction. The experimental design incorporated both untreated supernatants and lysis buffer-treated cells to establish baseline and peak release values, respectively. The final toxicity index was derived by calculating the ratio of secreted (extracellular) LDH to the cumulative (total) LDH pool, the latter being the sum of the medium and lysate fractions.

### 2.5 Cell Proliferation Assay

Cell proliferation was measured by incorporation of bromo-deoxy-uridine (BrDU), using the BrdU Cell proliferation assay kit purchased (Cell Signaling Technology, Danvers, MA, USA; Cat. No. 6813). RES 186 and RES 259 were plated at a density of 10.000 cells/well in 96-well plates. Background wells without BrdU incorporation (no-BrdU controls) were included to estimate the background signal. BrdU was spiked in the last 20 h (O/N) of the incubation period. The assay was conducted in accordance with the manufacturer’s instructions. The kit detects 5-bromo-2′-deoxyuridine (BrdU) incorporated into cellular DNA during cell proliferation using an anti-BrdU antibody. When cells are cultured in a medium containing BrdU, this thymidine analogue is incorporated into the newly synthesized DNA of proliferating cells. After removing the medium, the cells are fixed, and the DNA is denatured using a fixation/denaturation solution. A mouse monoclonal anti-BrdU antibody (Cell Signaling Technology, Danvers, MA, USA; Cat. No. 94079) is then added at a 1:100 dilution to detect the incorporated BrdU (DNA denaturation is necessary to improve the accessibility of the incorporated BrdU to the detection antibody). The anti-mouse IgG-HRP conjugated antibody (Cell Signaling Technology, Danvers, MA, USA; Cat. No. 34709S) is then used at a 1:100 dilution to recognize the bound detection antibody. The HRP substrate (3,3′,5,5′-Tetramethylbenzidine TMBMB, Cell Signaling Technology, Danvers, MA, USA; Cat. No. 7004P4) is added to detect the color. Utilizing a PerkinElmer photometer Victor 4 (Waltham, MA, USA), we monitored the cell proliferation by recording absorbance levels at a wavelength of 450 nm.

### 2.6 Cell Migration Assay

To evaluate the potential effect of Mebendazole on cell migration and correlated tumor invasion, a cell migration assay was conducted. RES 186 and RES 259 were plated in the upper part of the special chamber at a density of 2 × 10^4^. Transwells with porous membrane, with pores of 8.0 μm (Corning, New York, NY, USA; Cat. No. 3470) were used. Cells were plated in starvation conditions (serum-free medium). In the lower part of the well, we placed medium with 10% of serum provided with the treatments under investigation (MBZ 100 nM–1 μM), or 10% of serum alone. Cells were allowed to migrate at 37°C in 5% CO_2_ for 18 h–20 h. After, cells were rinsed with PBS 10× (pH 4.2–4.6; Sigma-Aldrich, St. Louis, MO, USA; Cat. No D 1283) with Ca^2+^ and Mg^2+^ and fixed with 4% formaldehyde. After washing in PBS with Ca^2+^ and Mg^2+^ and Methanol 100%, cells were coloured with Giemsa, previously diluted 20-fold in distilled water, for 45 min. Then, cells were washed with distilled and acidulated water and the well membrane was fixed on a slide. Cells that did not migrate were removed from the top using cotton wool (cotton bud). Stained membranes were mounted on a microscope slide and images were captured with a microscope Zeiss Axiovert 25 (Zeiss Axiofot, East Lyme, CT, USA). Migrated cells were quantified by manual counting under the microscope. For each condition, two representative fields out of four were selected from images acquired at 20× and 40× magnification in duplicate. The number of migrated cells was manually counted, and the mean value from the selected fields was calculated. Results were expressed as the percentage of migrated cells relative to the control condition.

### 2.7 Western Immunoblot Analysis

Both RES 259 and RES 186 were treated with MBZ 10 nM and 1–10 μM for 48 h. All the treatments were administered in culture medium supplemented with 1% fetal bovine serum (FBS) and 1% penicillin–streptomycin. At the conclusion of the experiment, cells were collected by scraping in 1× PBS (pH 7.4) without Ca^2+^ and Mg^2+^ and centrifuged at 1200 rpm for 5 min (Eppendorf, Hamburg, Germany; Cat. No. 5702R). Cells were subsequently lysed using RIPA buffer prepared with 1 mM EDTA [1 mM EDTA (Cat. No.: E7889), 150 mM NaCl (Cat. No.: S9888), 1% igepal (Cat. No.: I3021), 0.5% sodium deoxycholate (Cat. No.: D-6750), 50 mM Tris–HCl, pH 8.0 (Cat. No.: T-3038) (Sigma-Aldrich, St. Louis, MO, USA), and 0.1% sodium dodecyl sulfate, SDS, (Cat. No.:1610416; Bio-Rad, Hercules, CA, USA)] containing protease inhibitor cocktail diluted 1:250 (Cat. No: P8340; Sigma-Aldrich, St. Louis, MO, USA) and sodium orthovanadate, phosphatase inhibitor diluted 1:10 (Na_3_VO_4_, Cat. No. S6508, Sigma-Aldrich, Merck KGaA, St. Louis, MO, USA). Following lysis, cell lysates were centrifuged at 13,000 rpm for 10 min at 4°C, and the supernatants were stored at −80°C. Protein concentrations were determined using the Bradford assay. For each sample, 30 μg of protein were combined with 4× Bolt™ LDS Sample Buffer (Cat. No.: B0007; Novex, Carlsbad, CA, USA) and 10× Bolt™ Sample Reducing Agent (Cat. No.: B0009; Novex, Carlsbad, CA, USA), then heated at 95°C for 5 min. The prepared samples were subsequently loaded onto precast NuPAGE™ 4–12% Bis-Tris mini gels (1.0–1.5 mm, Invitrogen, Carlsbad, CA, USA) for electrophoresis (). For molecular weight determination, Precision Plus Protein™ All Blue Prestained Protein Standards (Bio-Rad, Hercules, CA, USA; Cat. No.: 161-0373) were used, with bands ranging from 10 to 250 kDa (10, 15, 20, 25, 37, 50, 75, 100, 150, and 250 kDa). In addition, SeeBlue™ Plus2 Pre-Stained Standard (Invitrogen, Life Technologies, Carlsbad, CA, USA, Cat. No.: LC5925) was employed, containing bands from 4 to 250 kDa (pre-stained at 4, 6, 8, 10, 15, 20, 30, 40, 50, 60, 70, 80, 100, 120, 150, 180, 220, and 250 kDa).

Proteins were first separated by electrophoresis, initially at 200 V for 5 min, followed by 150 V for 60 min. They were then transferred onto a PVDF membrane (Invitrogen, Carlsbad, CA, USA) using the iBlot™ 2 Gel Transfer Device (Invitrogen, Carlsbad, CA, USA). Various primary antibodies were tested, all prepared in iBind™ Flex Solution (Invitrogen, Carlsbad, CA, USA), which also served as the blocking solution. Membranes were incubated with the primary antibodies for 2 h at room temperature or overnight at 4°C with gentle agitation (details in Table 1). The following day, the membranes were washed twice with TBS-T before incubation with the secondary antibody, also diluted in Flex Solution, for 1 h. After two additional TBS-T washes, protein bands were visualized using chemiluminescence. Detection was performed on a ChemiDoc™ XRS system (Bio-Rad) after applying ECL reagents (SuperSignal™ West Pico PLUS Chemiluminescent Substrate, Thermo Scientific™, Rockford, IL, USA, or Pierce™ ECL Western Blotting Substrate). Densitometric quantification of Western blot bands was performed using Image Lab software (version 6.0.1; Bio-Rad). The intensity of each band was first normalized to β-actin, which served as the housekeeping control to account for loading variability. For phosphorylated proteins, the ratio between the phosphorylated form and the corresponding total protein was calculated. Both phosphorylated and total protein signals were independently normalized to β-actin before calculating the ratio, thus ensuring that the values accurately reflected the relative phosphorylation status corrected for protein loading.

Primary and secondary antibodies, the related dilutions are reported in the table below.

**Table 1:** Antibody information used for Western immunoblotting analysis.

Antibody	Diluition	Producer	Catalog Number	Clone	Host	MW
β-tubulin III	1:1000	Sigma	#T8578-100ul	TUJ1	Mouse mAb	~50–55 kDa
RAS GAP	1:500	BD Bioscience	#15895219	44/RASGAP	Mouse mAb	~120–135 kDa
p-MEK (Ser 217/221)	1:1000	Cell signaling	#9154	D2E9	Rabbit mAb	~45 kDa
Total MEK	1:1000	Cell signaling	#9122	L38C12	Mouse mAb	~45 kDa
p-Erk1/2 (Thr202/Tyr204)	1:1000	Cell signaling	#9101	20G11	Rabbit mAb	ERK1: ~44 kDa; ERK2: ~42 kDa
Total Erk 1/2	1:1000	Cell signaling	#9102	137F5	Rabbit mAb	ERK1: ~44 kDa; ERK2: ~42 kDa
p-CREB (Ser 133)	1:1000	ThermoFisher	#PA1-4619	Polyclonal (pAb)	Rabbit	~43 kDa
Total Creb	1:500	ThermoFisher	#PA1-850	Polyclonal (pAb)	Rabbit	~43 kDa
IKB-α	1:1000	Santa Cruz	#SC-371	C-21	Rabbit pAb	~36 kDa
p 21	1:500	Cell signaling	#2947	12D1	Rabbit mAb	~21 kDa
NFκB	1:1000	Cell signaling	#8242	D14E12	Rabbit mAb	~65 kDa
Claeved caspase 3	1:1000	Cell signaling	#9664	5A1E	Rabbit mAb	Cleaved: ~17/19 kDa
LC3A	1:250	Novus	#NB100-2331	Polyclonal (pAb)	Rabbit	LC3-I: ~16 kDa; LC3-II: ~14 kDa
p-p53 (ser 46)	1:1000	Cell signaling	#2521	Polyclonal (pAb)	Rabbit	~53 kDa
Beclin	1:1000	Novus	#NB500-249	Polyclonal (pAb)	Rabbit	~60 kDa
N-cadherin	1:1000	BD Bioscience	#610920	32/N-Cadherin	Mouse mAb	~130 kDa
β-actin	1:1000	Sigma	#A 5316	AC-15	Mouse mAb	~42 kDa
Anti-mouse	1:3000	Sigma	#A 3682	Polyclonal (pAb)	Goat	Detects mouse IgG
Anti-rabbit	1:15,000	JacksonImmuno Research	#111-035-045	Polyclonal (pAb)	Goat	Detects rabbit IgG

### 2.8 Statistical Analyses

GraphPad Prism (v. 7.4) served as the analytical platform for all statistical evaluations. Inter-group comparisons were scrutinized via one-way ANOVA followed by Dunnett’s multiple comparison test. We adopted a standard confidence level, where results were deemed statistically robust only if the *p*-value was less than 0.05.

## 3 Results

### 3.1 Effects of MBZ on Cell Metabolic Activity, Cytotoxicity and Cell Proliferation

To investigate the potential antitumor activity of MBZ in pediatric low-grade gliomas (pLGG), we selected the RES186 and RES259 cell lines—two clinically and biologically distinct models widely used in preclinical research. Recent pharmacological studies have confirmed their relevance; for instance, both lines respond to mTOR inhibitors with a reduction in proliferation and migration, underscoring their utility in evaluating therapeutic interventions in pLGG [45].

First experiments were conducted to evaluate antiproliferative effects of MBZ doses ranging from 1 nM to 100 μM on RES 259 and RES 186 cell lines. This wide concentration range was selected to investigate both low-dose effects, potentially relevant to clinically achievable exposures, and higher doses that allow exploration of the full pharmacological profile of MBZ, including potential off-target mechanisms.

**Figure 1:**
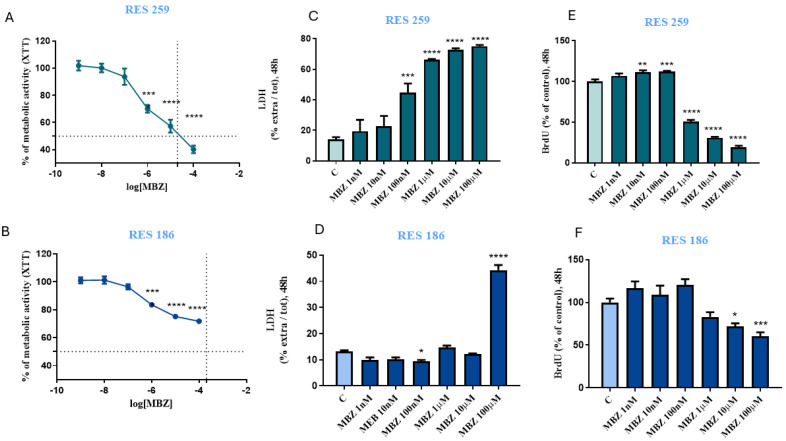
Evaluation of MBZ toxicity in RES 259 and RES 186. Effect of Mebendazole, ranging from 1 nM to 100 μM, on cell metabolic activity by XTT assay (**A**,**B**), on cellular damage by extracellular/total LDH ratio measurement (**C**,**D**) on proliferation by BrdU incorporation (**E**,**F**). 48 h-experiments were carried out. Data are expressed as a percentage relative to the untreated cells (control = 100%) and are means ± standard error (SEM). One-way ANOVA analysis, followed by Dunnett’s post-test, was conducted. *p < 0.05, **p < 0.01, ***p < 0.001, ****p < 0.0001. All *p*-values were calculated versus the control sample.

After 48 h-treatment, a dose-dependent reduction in metabolic activity was observed. MBZ significantly reduced metabolic activity by approximately 40% at a dose of 1 μM, up to a reduction greater than 50% with 100 μM in RES 259; while in the RES 186 cell line, a significant decrease in metabolic activity was observed between 1 μM and 100 μM, leading to a reduction of about 20%. In RES259 cells, the IC_50_ was reached at the highest micromolar doses tested, whereas in RES186 cells, the 50% reduction threshold was not achieved within the tested concentration range (Fig. 1A,B). Consistently, total protein content assays confirmed a parallel decrease in the number of viable cells, further supporting the reduction in cell viability induced by MBZ treatment (Supplementary Fig. S1).

After 48 h of exposure, MBZ increased LDH release, expressed as a percentage of extracellular LDH relative to total LDH. In RES 259, there was a significant dose-dependent increase, while in RES 186, the increase in LDH release was significant only at the 100 μM dose (Fig. 1C,D).

Finally, the proliferation of newly synthesized cells was evaluated through the incorporation of BrdU at 48 h. Both cell lines initially showed a slightly increased proliferation at doses between 1 nM and 100 nM, followed by a dose-dependent and significant reduction at higher doses (Fig. 1E,F).

### 3.2 Effect of MBZ on the MAPK Pathway

**Figure 2:**
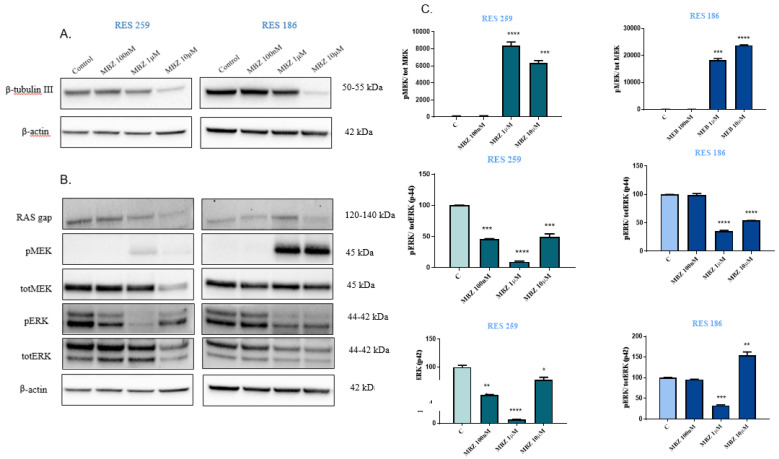
Western blot analysis in RES 259 and RES 186 of the main proteins involved in the MAPK pathway after 48 h of the following treatments: Lane 1, control; Lane 2, Mebendazole 100 nM; Lane 3, Mebendazole 1 μM; Lane 4, Mebendazole 10 μM. (**A**,**B**). Panel (**C**) shows the quantification of phosphorylated proteins on total (pMEK/MEK tot; pERK/ERK tot). Data are means ± SEM, and were analyzed by one-way ANOVA, followed by Dunnett’s post-test. *p < 0.05, **p < 0.01, ***p < 0.001, ****p < 0.0001. For every protein set, β-actin is reported as the normalizer gene.

The MAPK pathway is involved in pLGG and it has been proven that MBZ interferes with this pathway by altering the expression of key factors [46]. To explore this further, we investigated the protein expression of several crucial components of this pathway through Western blot (Fig. 2).

The mechanism of action of this drug was initially confirmed by assessing the expression of β-tubulin III, the MBZ primary target (Fig. 2A). Notably, β-tubulin levels were found to decrease in a dose-dependent manner, with a marked reduction observed at the highest dose (10 μM) for both cell lines (Supplementary Fig. S2).

The RAS protein, located upstream of the MAPK pathway, was included in the analysis. RAS expression decreased significantly and in a dose-dependent manner in RES 259 cells. In contrast, RES 186 cells exhibited a significant increase in RAS expression at a 1 μM dose, followed by a drastic reduction at the highest dose of 10 μM (Fig. 2B). We also examined both the phosphorylated and total forms of the MEK kinase, along with their ratio. The phosphorylated form increased at the 1 μM dose in RES 259 and at both 1 and 10 μM in RES 186. The total form decreased at 10 μM in RES 259, whereas in RES 186, no significant effect was reported. The pMEK/totalMEK ratio significantly increases at the two doses, 1 μM and 10 μM, in both RES 259 and RES 186 (Fig. 2B,C). Next, we assessed the main effector protein of the MAPK pathway, ERK, specifically its two isoforms, ERK1 (44kDa) and ERK2 (42kDa), in both phosphorylated and total forms. Quite unexpectedly, both pERK isoforms (p44 and p42) showed a significant reduction in expression of more than 50% in RES 259 and RES 186. The two total ERK isoforms also had a similar pattern, with a significant decrease at 10 μM in both cell lines. The pERK/total ERK ratio of both isoforms showed a similar trend with a significant overall reduction at the highest doses in both RES 259 and RES 186 (Fig. 2B,C).

### 3.3 Effects of MBZ on Apoptosis and Autophagy Pathways

**Figure 3:**
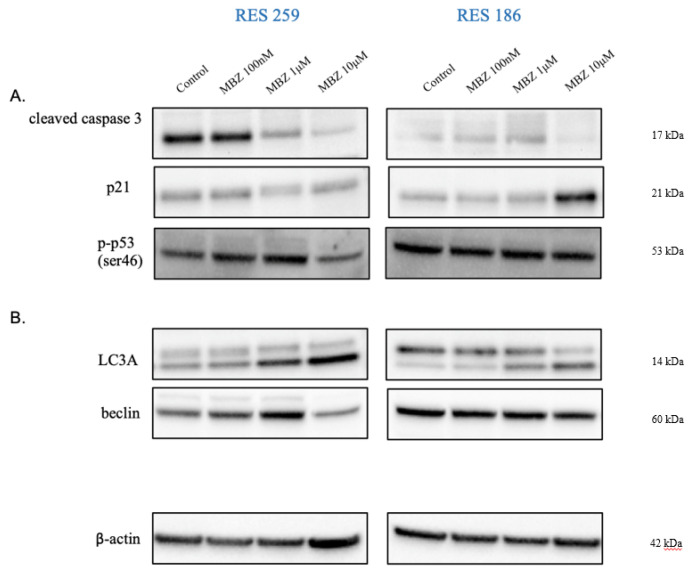
MBZ modulates apoptosis- and autophagy-related proteins in RES 259 and RES 186 cells. The involvement of mebendazole (MBZ) in regulating key proteins associated with apoptosis (**A**) and autophagy (**B**) was evaluated in RES 259 and RES 186 cell lines. Cells were treated for 48 h with increasing concentrations of MBZ as follows: Lane 1, untreated control; Lane 2, 100 nM MBZ; Lane 3, 1 μM MBZ; Lane 4, 10 μM MBZ. For every protein set, β-actin is reported as the normalizer gene.

According to the literature, MBZ plays a role in regulating apoptosis, prompting an investigation into several proteins involved in this process [47]. The expression of executioner caspase 3 was evaluated, specifically its cleaved form (p17). This showed a dose-dependent reduction in RES 259, while there was a significant increase at the 100 nM and 1 μM doses, followed by a decrease at the 10 μM dose in RES 186. In addition, the cyclin-dependent kinase inhibitor p21 was studied. p21 expressed a gradual reduction in RES 259, while there was a substantial and significant increase at a 10 μM dose in RES 186. The transcription factor p53, known for its role in inducing apoptosis, was also observed in its phosphorylated form. In RES 259, p-p53 (ser46) increased at the first two doses but decreased at the highest dose. In RES 186, p-p53, no significant modification was reported (Fig. 3A and Fig. S3A).

Autophagy, another mechanism through which MBZ exerts its anti-tumor effects [48], was also explored. Beclin, a key protein in the autophagic process, showed an increase followed by a significant decrease in RES 259, while in RES 186, its level remained unchanged until a marked reduction at 10 μM. Lastly, the autophagy-related protein LC3A was investigated. LC3A expression increased significantly in a dose-dependent manner in both cell lines (Fig. 3B and Fig. S3B).

### 3.4 Effect of MBZ on Migration of RES259 and RES186 Cell Lines

**Figure 4:**
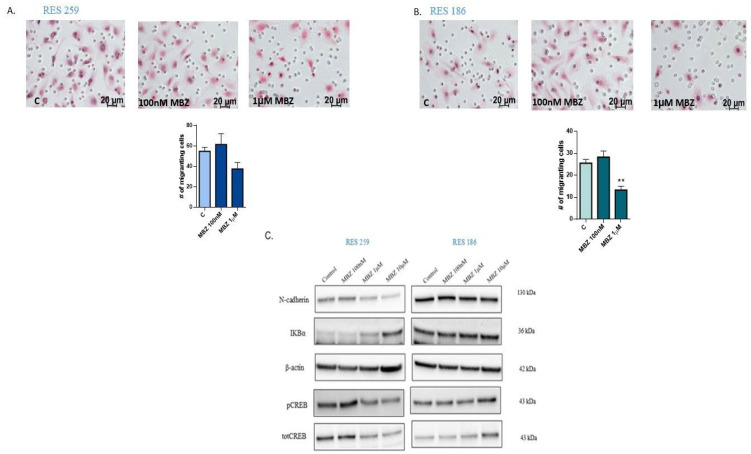
Effect of MBZ on the migration of RES 259 and RES 186 and potential role in reducing tumor invasiveness. (**A**,**B**) show untreated cells, cells treated with 100 nM Mebendazole and cells treated with 1 μM Mebendazole. The cell number count was calculated as mean ± SEM and one-way ANOVA analysis was performed, followed by Dunnett’s post-test. **p < 0.01. (**C**) Western blot analysis in RES 259 and RES 186 of some proteins involved in the EMT mechanism after 48 h of the following treatments: Lane 1, control; Lane 2, Mebendazole 100 nM; Lane 3, Mebendazole 1 μM; Lane 4, Mebendazole 10 μM. For every protein set, β-actin is reported as the normalizer gene.

As regards cell migration, cells were exposed to 100 nM and 1 μM doses of Mebendazole for 20 h. MBZ was able to inhibit the migration in both cell lines, significantly reducing the number of migrating cells compared to the vehicle in RES 259 (Fig. 4A). Instead, RES 186 cells showed a reduction in migration, although the decrease did not reach statistical significance (Fig. 4B). To elucidate the mechanism behind MBZ’s inhibition of migration, the expression of proteins involved in EMT was evaluated, focusing on N-cadherin, IKBα and CREB.

N-cadherin showed a dose-dependent and significant reduction in expression in RES 259, while in RES, no significant effect was reported (Fig. 4 and Fig. S4A). IKBα expression increased significantly and progressively with higher doses in RES259, while no significant effect was reported in RES186 (Fig. 4B and Fig. S4B).

Finally, we analyzed the transcription factor CREB in both its phosphorylated and total forms. The total form (Fig. 4C and Fig. S4C) underwent a dose-dependent and highly significant reduction at the highest dose in RES 259 and a dose-dependent and significant increase in RES 186. The pCREB/total CREB ratio showed opposite effects: an increase at the lowest dose and remained unchanged at the other doses in RES 259, while it showed a significant and dose-dependent reduction in RES 186 (Fig. 4C and Fig. S4D), as a result of the fact that pCREB (Fig. 4C and Fig. S4E), showed an increased expression at 100 nM and a significant reduction with an increasing dose in RES 259. No significant effect was reported in RES 186.

### 3.5 Comparison with the Gold Standard: Vinblastine

The efficacy of Vinblastine was tested on the two cell lines to assess its ability to stabilize or reduce tumor growth and to determine the minimum effective dose.

**Figure 5:**
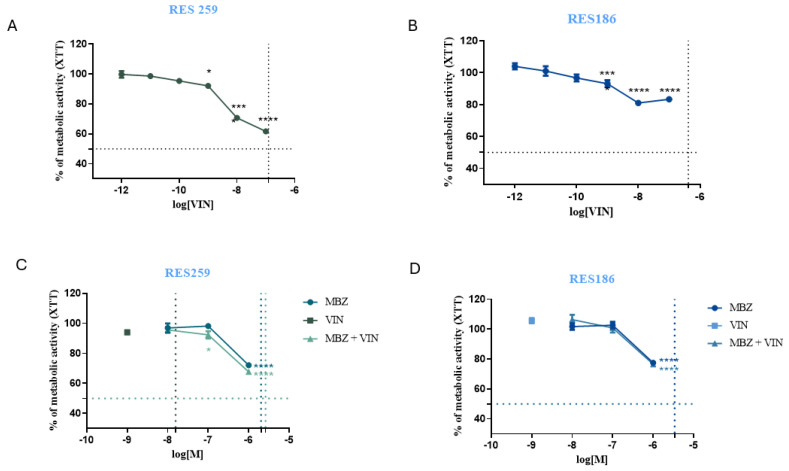
Evaluation of Vinblastine alone or in combination with MBZ on cell survival in RES 259 and RES 186. Cell viability was assessed after 48 h of treatment by XTT assay and expressed as a percentage relative to untreated cells (control = 100%). (**A**,**B**) Effect of vinblastine (VIN) alone, tested at concentrations ranging from 1 pM to 100 nM, on the metabolic activity of RES 259 (**A**) and RES 186 (**B**) cells by XTT assay. (**C**,**D**) Effect of vinblastine (1 nM) in combination with mebendazole (MBZ; 100 nM or 1 μM) on RES 259 (**C**) and RES 186 (**D**) cells. The treatment conditions included: control, MBZ 100 nM, MBZ 1 μM, MBZ 100 nM + VIN 1 nM, MBZ 1 μM + VIN 1 nM, and VIN 1 nM alone. Data are presented as mean ± SEM. Statistical analysis was performed using one-way ANOVA followed by Dunnett’s post hoc test. *p < 0.05, ***p < 0.001, ****p < 0.0001.

The lowest doses, in the pM range, had no impact on cell viability, while the drug began to exhibit activity at nM concentrations. At 100 nM, vinblastine significantly reduced cell viability by nearly 50% in RES 259 cells, and to a lesser, but still significant, extent in RES 186 cells. IC_50_ values were calculated from the corresponding dose–response curves. When Vinblastine was used alone, the IC_50_ was reached only at the highest dose tested and exclusively in RES259 cells, whereas in RES 186, the 50% reduction threshold was not achieved within the tested concentration range (Fig. 5A,B).

To compare the effects of Vinblastine versus MBZ and evaluate their potential synergistic or combination effects, the 1 nM dose of Vinblastine was selected. This dose, though lower than the minimum effective concentration, still showed a modest but significant inhibition of cell viability, making it suitable for use in combination with MBZ. The dose range chosen for MBZ, based on prior results, was from 10 nM to 1 μM, as these concentrations demonstrated antiproliferative effects on both RES 259 and RES 186. Then, the cells were treated with MBZ at doses of 10 nM, 100 nM and 1 μM, either alone, in combination with 1 nM of Vinblastine, or with Vinblastine alone at 1 nM. At the same time, the response of the cells to MBZ alone, Vinblastine alone, and their association was studied in order to research a possible enhancement of their anti-tumor activity.

Cell metabolic activity was measured at 48 h in RES259 and RES186. The association was clearly more effective than Vinblastine alone, while compared to MBZ monotherapy, the overall trend was very similar, although the IC_50_ appeared to be reached slightly earlier in the combined treatment (Fig. 5C,D).

Parallelly, the total protein content was measured at 48 h in cells treated with Vinblastine alone, MBZ alone, and their combination, showing a significant reduction in the combination group compared to either single treatment (Supplementary Fig. S5).

**Figure 6:**
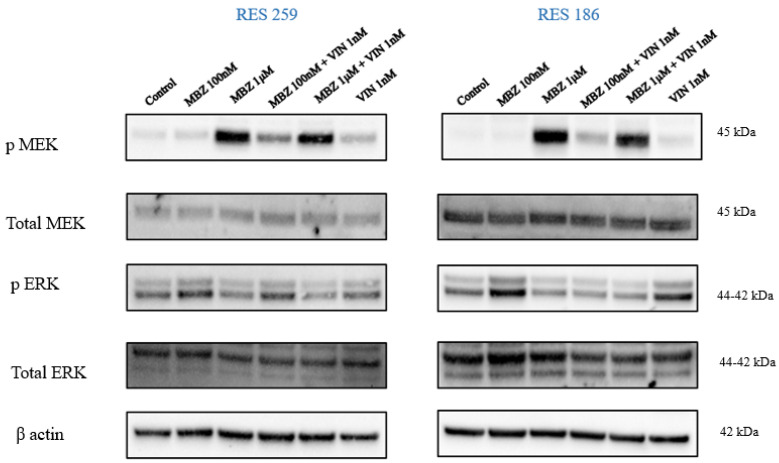
Western blot analysis in RES 259 and RES 186 of some proteins involved in the MAPK pathway after 48 h of the following treatments: Lane 1, control; Lane 2, Mebendazole 100 nM; Lane 3, Mebendazole 1 μM; Lane 4, Mebendazole 100 nM and Vinblastine 1 nM; Lane 5, Mebendazole 1 μM and Vinblastine 1 nM; Lane 6, Vinblastine 1 nM.

Similar results were obtained in the MAP Kinase pathway study. Neither in MEK nor in ERK protein levels did the combination treatment significantly modify the increase reported by MBZ alone (Fig. 6 and Fig. S6).

## 4 Discussion

In the present study, we conducted an extensive series of experiments to evaluate the potential repositioning of MBZ in two distinct pediatric low-grade glioma cell lines. After 48 h, we observed cytotoxicity and inhibition of cell growth in both RES 259 and RES 186, with slight differences between the two. MBZ demonstrated an antiproliferative effect in both cell lines, although with varying potency.

We selected two pediatric low-grade glioma (pLGG) cell lines, RES186 and RES259, to capture intra-tumoral heterogeneity. RES259, derived from a diffuse astrocytoma (WHO grade II), exhibits notable stem-like attributes, including higher proliferation and self-renewal capacity in long-term culture, as evidenced by a recent stem-profiling study of pediatric glioma lines [49,50]. On the other hand, RES186, from a pilocytic astrocytoma (WHO grade I), is characterized by atypical genetic alterations such as PTEN deletion and TP53 loss of heterozygosity, suggestive of a transformation-prone phenotype *in vitro*, possibly less representative of canonical pLGG behavior [51]. By comparing drug responsiveness across these two divergent models—one with elevated stem-like and proliferative features (RES259), and the other with distinct tumor suppressor alterations (RES186)—we aimed to investigate the robustness of MBZ activity across molecularly diverse pLGG contexts. RES 259 and RES 186 exhibit differential sensitivity to MBZ: RES 259 cells show higher susceptibility and greater rates of cell death compared to RES 186. The differences are related to the reduction in cell viability, proliferation of newly formed cells, and the increase in the release of LDH. The differential response may be due to various factors, including intrinsic resistance mechanisms in the RES 186 line. MBZ primarily exerts its antitumor effect by disrupting microtubules. However, some studies, such as [52], suggest that cancer cells can develop mutations or alterations in tubulin, the building block of microtubules, leading to resistance. RES 186 cells might have microtubule-related mutations that make them less susceptible to MBZ-induced apoptosis, as one study identified mutations in the β-tubulin gene that confer resistance to microtubule-targeting agents in cancer cells [53]. In addition, RES 186 cells may activate alternative signaling pathways, such as the PI3K/AKT or MAPK pathways, which promote cell survival and reduce sensitivity to microtubule inhibition. Studies have shown that tumors can adapt by activating these pathways in response to treatment, making them more resistant to therapies that target specific cell structures like microtubules [54].

In particular, MBZ reduces RAS expression, thus impairing the ability of RAS to function properly in these pathways. In addition, MBZ increases MEK phosphorylation in RES 186 cells, likely as a compensatory response to the disruption of the RAS signaling pathway. MEK is a downstream effector in the RAS-RAF-MEK-ERK pathway, which regulates cell proliferation and survival. When MBZ reduces RAS expression or impairs its function, cells may activate compensatory mechanisms to maintain signaling through the pathway. This increased phosphorylation may represent the cell’s effort to bypass the block in the pathway caused by MBZ, especially in cancer cells, where survival signaling is critical for growth [50,55,56].

ERK is a key downstream effector of the MAPK pathway. Typically, MEK activation leads to ERK phosphorylation and activation. In our case, despite increased MEK phosphorylation, ERK phosphorylation is reduced. This could imply that while MEK is being activated, there is a disruption or inhibition in the signaling from MEK to ERK, potentially due to the reduced RAS activity or other factors affecting the pathway.

Since ERK is known to phosphorylate and activate CREB indirectly [57], the decrease in ERK phosphorylation caused by MBZ likely results in reduced CREB activation. CREB is a crucial transcription factor that, when phosphorylated, activates genes involved in cell survival, proliferation and differentiation. This disruption in CREB activation could affect various cellular processes, including those related to tumor growth, survival and migration [58].

Overall, MBZ appears to interfere with the MAPK signaling pathway by reducing RAS expression and disrupting the normal flow of activation through the pathway. This results in increased MEK activation but decreased ERK activation, which may contribute to its effects on tumor cell growth and survival.

Inhibition of this pathway can make tumor cells more susceptible to apoptosis. In our experiments, cleaved caspase, p21 and LC3A are modified by MBZ. The differences observed in apoptotic and autophagic responses are likely linked to the unique molecular characteristics of each line and their individual response to cellular stress induced by MBZ.

In the RES 259 cell line, treatment with MBZ at lower concentrations (100 nM and 1 μM) failed to induce apoptosis. This was confirmed by the reduction in cleaved caspase-3 and p21 levels, both key markers of apoptosis. Caspase-3 is an executioner caspase that cleaves cellular components necessary for the final stages of apoptosis. Its suppression indicates that the apoptotic pathway, particularly the intrinsic mitochondrial pathway, might be blocked or less activated [59].

Similarly, p21, a cyclin-dependent kinase inhibitor that mediates cell cycle arrest and can promote apoptosis in a p53-dependent manner, was also reduced. Since p21 is a well-established downstream effector of the MAPK/ERK pathway and a key regulator of cell cycle progression in gliomas, it was included in our analysis to evaluate whether MBZ treatment disrupts cell cycle control mechanisms. The relevance of this marker is further supported by recent findings showing that p21 participates in the EGFR/AKT/ERK/p21 signaling axis in glioblastoma, where it modulates cancer progression and influences therapeutic resistance [60]. This suggests that MBZ does not initiate a typical DNA-damage-induced apoptotic response in RES 259 at these doses.

However, there was an upregulation of LC3A and beclin, two key proteins involved in the autophagy process. Beclin is an essential regulator of autophagy initiation, and LC3 (particularly its conversion from LC3-I to LC3-II) is involved in the elongation of autophagosomes. The increase in these markers suggests that instead of triggering cell death via apoptosis, MBZ drives RES 259 cells towards autophagy, which is often a survival mechanism under stress conditions, especially when apoptosis is not engaged [61].

Additionally, the rise in p53 phosphorylation suggests that Mebendazole induces cellular stress and DNA damage in RES 259 cells. Phosphorylated p53 acts as a transcription factor that can promote both apoptosis and autophagy [62]. We specifically analyzed p53 phosphorylation at Ser46 because this post-translational modification is known to act as a molecular switch that directs p53 activity toward the transcription of pro-apoptotic target genes and the induction of mitochondrial apoptosis, particularly in the presence of severe DNA damage [63]. Although the absence of total p53 reduces the strength of interpretation in absolute terms, normalization to β-actin provides a reliable comparison across conditions, and the findings gain significance when considered together with the lack of apoptotic markers and the presence of an autophagic response. This suggests that MBZ does not activate canonical p53-mediated apoptosis but may preferentially promote p53-mediated autophagy, potentially as an initial survival response to the drug.

In contrast, the RES 186 cell line shows activation of both apoptotic and autophagic pathways after treatment with MBZ. Autophagy is also upregulated in RES 186, as seen by the moderate increase in LC3A and beclin. This autophagic response is less pronounced than in RES 259, suggesting that while RES 186 cells do engage in autophagy, it may be secondary to the apoptotic response. Autophagy in this context might serve a more cytoprotective role, allowing cells to manage stress or damaged organelles, but it is not the dominant survival mechanism as it appears to be in RES 259.

At higher concentrations (10 μM), both cell lines exhibit a markedly different response, likely due to non-specific toxic effects or extensive cellular damage. At this concentration, MBZ may overwhelm cellular repair and survival mechanisms, leading to cell death that is less dependent on the classical pathways of apoptosis or autophagy. In fact, at high doses, drugs like MBZ can cause excessive damage to cellular structures, induce mitochondrial dysfunction, or generate excessive reactive oxygen species (ROS), leading to necrosis or other forms of cell death that are more chaotic and uncontrolled compared to apoptosis [64]. This non-specific damage can also disrupt cellular signaling pathways, leading to variable effects that are not neatly categorized as apoptotic or autophagic responses.

Pediatric pilocytic astrocytoma is traditionally not regarded as a highly infiltrative or invasive tumor, as it generally exhibits slow growth and a well-circumscribed—though not invariably sharply defined—architecture, and is frequently curable through gross total resection. Nevertheless, despite the absence of metastatic dissemination, microscopic infiltration of the adjacent brain parenchyma can occur, with tumor cells extending beyond the apparent radiographic or surgical margins [65]. This infiltrative propensity contributes to the challenge of achieving complete surgical excision and is a recognized factor associated with local recurrence [66]. Likewise, although pLGG are classified as non-metastatic and low-grade, they may still demonstrate locally infiltrative growth within the brain parenchyma, thereby complicating attempts at complete resection. Such infiltrative behavior—while not invasive in the classical malignant sense—nonetheless contributes to recurrence and neurological morbidity [13]. In this context, *in vitro* migration assays represent a tractable and informative model for evaluating tumor cell motility, serving as a surrogate measure of infiltrative potential. This approach is particularly relevant because alterations in migratory capacity may reflect underlying changes in cellular phenotype or tumor–microenvironment interactions that influence disease progression [67].

In light of this evidence, and given the interest expressed by other researchers [68,69] in this aspect, we evaluated whether MBZ might also exert an effect on reducing the invasive potential of the cells.

On RES 259 and RES 186, MBZ significantly reduces migration, especially at a dose of 1 μM in RES 259 cells. This reduction in migration is associated with changes in the expression of specific proteins involved in cell migration and the epithelial-mesenchymal transition. N-cadherin is a transmembrane protein that plays a crucial role in cell-cell adhesion and it is heavily involved in EMT, a process where epithelial cells acquire mesenchymal, migratory characteristics [70]. The reduction of N-cadherin expression in RES 259 and RES 186 cells after MBZ treatment directly inhibits migration. N-cadherin facilitates the detachment of tumor cells from the primary site and enhances their motility through decreased cell-cell adhesion and interaction with the extracellular matrix [71]. By reducing N-cadherin, MBZ likely increases cellular adhesion, making it more difficult for the tumor cells to detach and migrate. This also reduces their invasive capabilities, as N-cadherin is essential for interaction with mesenchymal tissues and other components necessary for metastasis.

IκBα is a protein that inhibits the NF-κB signaling pathway by sequestering NF-κB in the cytoplasm and preventing its translocation to the nucleus [72]. MBZ increases the expression of IκBα, which inhibits the activity of NF-κB. NF-κB normally upregulates genes that promote migration, such as Matrix Metalloproteinases (MMPs) and certain cytoskeletal proteins, which enable cells to degrade the ECM and facilitate migration. The inhibition of NF-κB by increased IκBα levels would therefore reduce MMP expression, limiting the ability of tumor cells to remodel their environment and migrate through tissues [73].

Finally, MBZ reduces CREB phosphorylation, thereby decreasing the transcriptional activity of CREB. In tumor cells, p-CREB supports migration by promoting the expression of genes that reorganize the cytoskeleton, such as actin-binding proteins and other molecules that facilitate cell movement. The reduction in p-CREB not only impairs the migratory machinery of the cell but also affects the survival pathways that are necessary for tumor cells to withstand the physical and metabolic stresses associated with migration [74]. Diminishing p-CREB levels, especially in RES259 MBZ, may also reduce the ability of tumor cells to undergo directional migration, thus inhibiting their movement towards metastatic sites.

Vinblastine and mebendazole share a common mechanism of action, targeting tubulin, a key component of microtubule formation. This interaction inhibits cell division and induces apoptosis. In the study by Kipper et al., the authors observed that the combination of MBZ and vinblastine resulted in a reduction in cell numbers. This finding positions the drug combination as a promising therapeutic approach, particularly for treating resistant gliomas that are less responsive to conventional treatments [75].

In our study, their combined use did not result in a synergistic effect. Microtubule dynamics are essential for mitosis and agents like MBZ and Vinblastine disrupt these processes by binding to tubulin. However, when two agents act on the same molecular target, the possibility of competition or saturation arises. In this case, the two drugs may compete for the same binding sites on tubulin, which could lead to a neutral or even antagonistic effect rather than potentiation of their individual actions. Plana et al. discuss the complex dynamics of synergy or lack thereof when multiple drugs with similar mechanisms are used in combination. The response to such combinations is highly variable and influenced by several factors, including the specific biological targets, the heterogeneity of the tumor, and the individual patient’s characteristics. This variability in outcomes highlights the importance of understanding whether drug interactions are truly synergistic, additive, or independently acting to achieve effective therapeutic strategies [76].

Finally, it is essential to acknowledge that the findings of this study are derived exclusively from *in vitro* experiments using immortalized cell lines. Although these models offer a controlled and reproducible environment, facilitating mechanistic investigations with reduced confounding variables, they do not fully recapitulate the complexity of native tissues or the physiological milieu of an intact organism. *In vitro* systems lack interactions with multiple cell types, extracellular matrix components, and dynamic *in vivo* conditions such as vascular perfusion and immune surveillance, and pharmacological responses observed *in vitro* may not accurately predict *in vivo* efficacy or toxicity. Nevertheless, these models enable the dissection of direct effects on specific cell populations and provide valuable insights into fundamental molecular processes. To address these inherent limitations, complementary *in vivo* studies are required.

## 5 Conclusion

In conclusion, this study highlights the potential of MBZ as a monotherapy for pediatric low-grade gliomas and raises important questions about the utility of combining microtubule-targeting agents. Further research is warranted to explore the molecular basis for MBZ’s broader efficacy and to investigate alternative combination strategies that may overcome the limitations observed when pairing it with Vinblastine. A deeper characterization of the molecular pathways modulated by MBZ, including downstream effectors and time-course analyses, would provide additional insight into its mechanism of action. In addition, further preclinical studies using different dosing schedules, drug ratios, and complementary *in vivo* models are needed to clarify whether a true synergistic effect between MBZ and Vinblastine can be established. These future investigations will contribute to optimizing therapeutic strategies and maximizing clinical benefit for patients with pediatric low-grade gliomas.

## Data Availability

Data will be made available on request.

## Supplementary Materials

The supplementary material is available online at https://www.techscience.com/doi/10.32604/or.2026.071074/s1.
