# Supplementary material for: Mebendazole Shows Antiproliferative and Antimigratory Effects in Paediatric Low-Grade Glioma Models
Source: Oncol Res. 2026 Jun 16;34(7):16. doi: 10.32604/or.2026.071074 (PMC13292026; doi:10.32604/or.2026.071074)
Supplement: Supplementary file 1 [file OncolRes-34-71074-s001.zip › TSP_OR_71074-s001.docx]

**Methods Supplementary**

1. ***Bradford Assay***

Bradford protein assay (Quick Start™ Bradford Protein Assay Kit, Biorad, Hercules, CA, USA, Cat. No. 5000006) was performed to quantify protein levels. RES 186 and RES 259 were treated and lysed in 100 μL Triton. In total, 10 μL of each treatment was used to calculate the amount of protein per well. BSA was chosen as the standard to develop a calibration curve ranging from 1 mg/mL to 0 mg/mL and the protein amount was measured as a function of absorbance at 570 nm using a microplate photometer (Victor 4, PerkinElmer, Waltham, MA, USA). Results were expressed as a total μg of proteins per well. Bradford assay was performed at 24h, 48h and 7 days of treatments.

**Figure Supplementary**


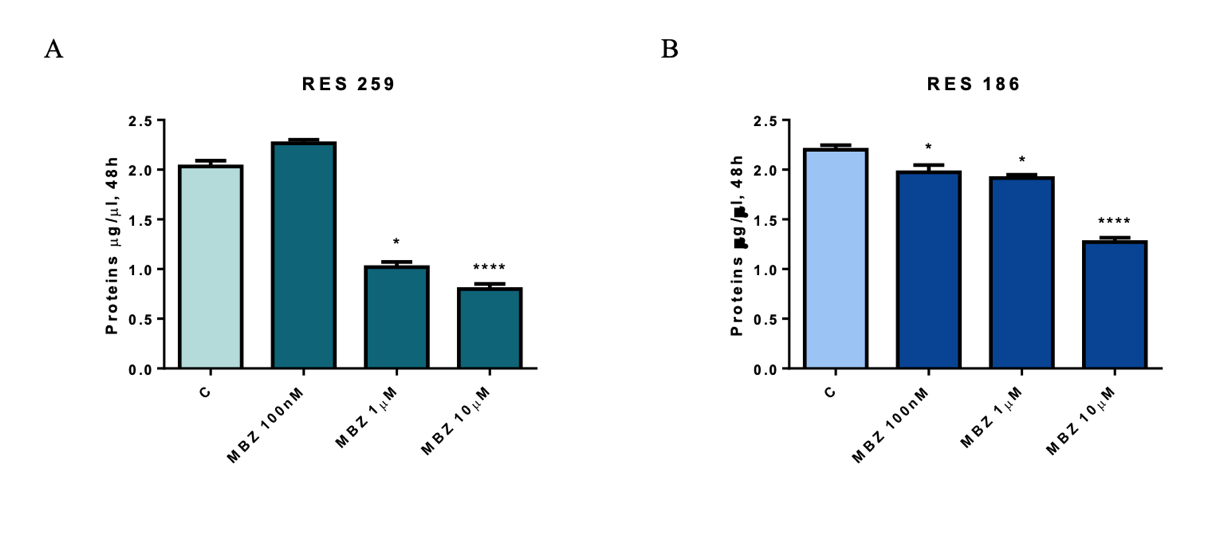


Supplementary Figure 1. Effect of mebendazole on protein content in RES 259 and RES 186 cell lines.
Protein amounts (µg/µL) were assessed after 48 h of treatment by the Bradford assay and expressed relative to untreated cells (control). **(A)** Effect of mebendazole (MBZ), tested at concentrations ranging from 100 nM to 10 µM, on protein content in RES 259 cells. **(B)** Effect of mebendazole (MBZ), tested at concentrations ranging from 100 nM to 10 µM, on protein content in RES 186 cells. Data are presented as mean ± SEM. Statistical analysis was performed using one-way ANOVA followed by Dunnett’s post hoc test. All p-values were calculated relative to the control sample. **p* < 0.05, *****p* < 0.0001.


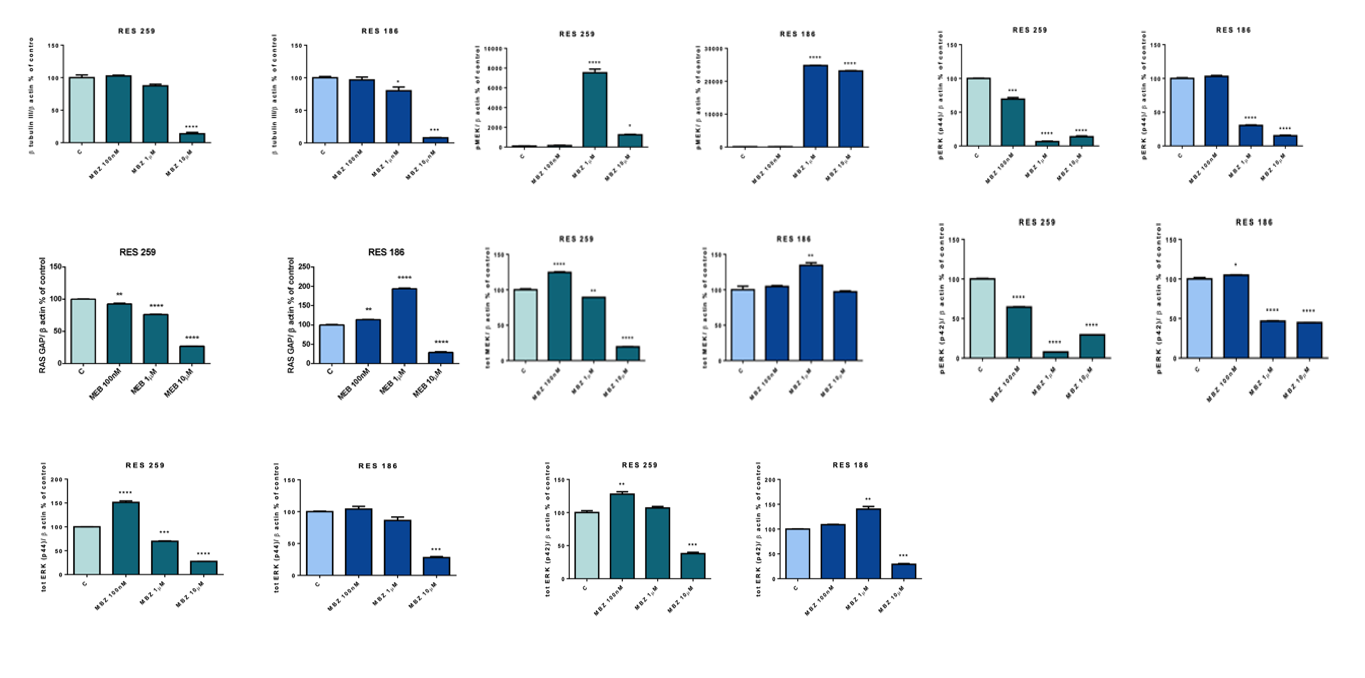


Supplementary Figure 2. Western blot analysis in RES 259 (green panels) and RES 186 (blue panels) of the main proteins involved in the MAPK pathway after 48 h of the following treatments: control, Mebendazole 100 nM, Mebendazole 1 μM, Mebendazole 10 μM. Data are means ± SEM, and were analyzed by one-way ANOVA, followed by Dunnett’s post-test. * *p* < 0.05, ** *p* < 0.001, *** *p* < 0.001, **** *p* < 0.0001. For every protein set, β-actin is reported as the normalizer gene.


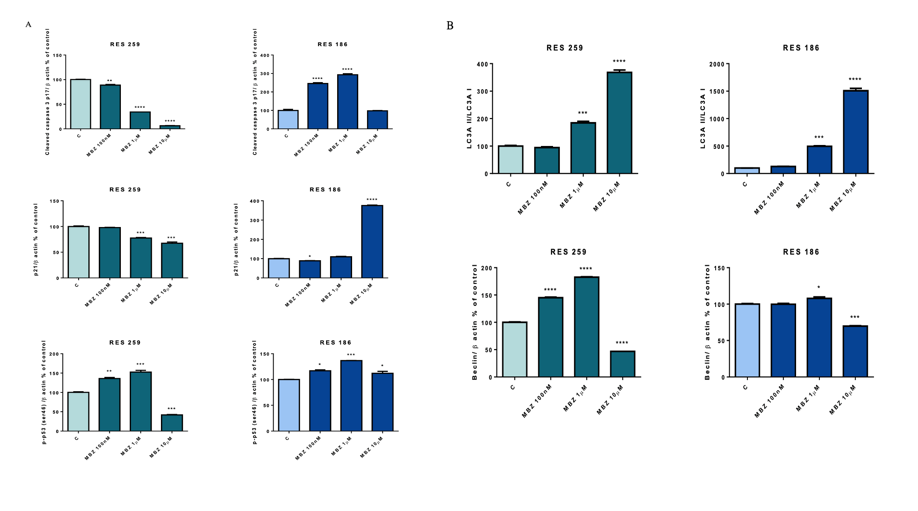


Supplementary Figure 3. The involvement of MBZ in regulating apoptosis and autophagy mechanisms was assessed after 48 h of treatment. (A) Apoptosis-related proteins in RES 259 (green panels) and RES 186 (blue panels) cells. (B) Autophagy-related proteins in RES 259 (green panels) and RES 186 (blue panels) cells.Cells were treated with: control, MBZ 100 nM, MBZ 1 µM, and MBZ 10 µM. Data are presented as mean ± SEM. For every protein set, β-actin was used as the normalizer. Statistical analysis was performed using one-way ANOVA followed by Dunnett’s post hoc test. **p* < 0.05; ** *p* < 0.01, ****p* < 0.001; *****p* < 0.0001.


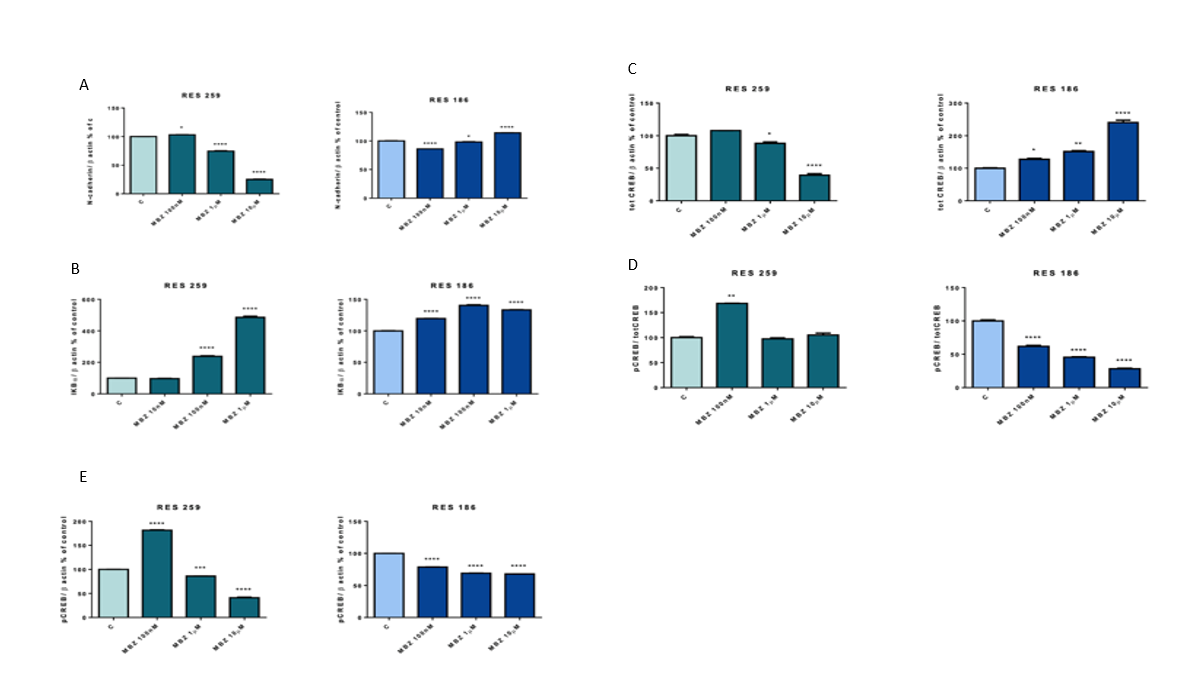


Supplementary Figure 4. Effect of MBZ on the migration of RES 259 and RES 186 and potential role in reducing tumor invasiveness, evaluating the protein level expression of some important proteins ( Panel A: N-Cadherin; Panel B: IKBα; Panel C: Total Creb; Panel D: ratio pCreb/Total Creb; Panel E: pCreb) involved in the EMT mechanism after 48 h of the following treatments: control, Mebendazole 100 nM, Mebendazole 1 μM, Mebendazole 10 μM* *p* < 0.05, ** *p* < 0.01, *** *p* < 0.001, **** *p* < 0.0001.For every protein set, β-actin is reported as the normalizer gene.

*
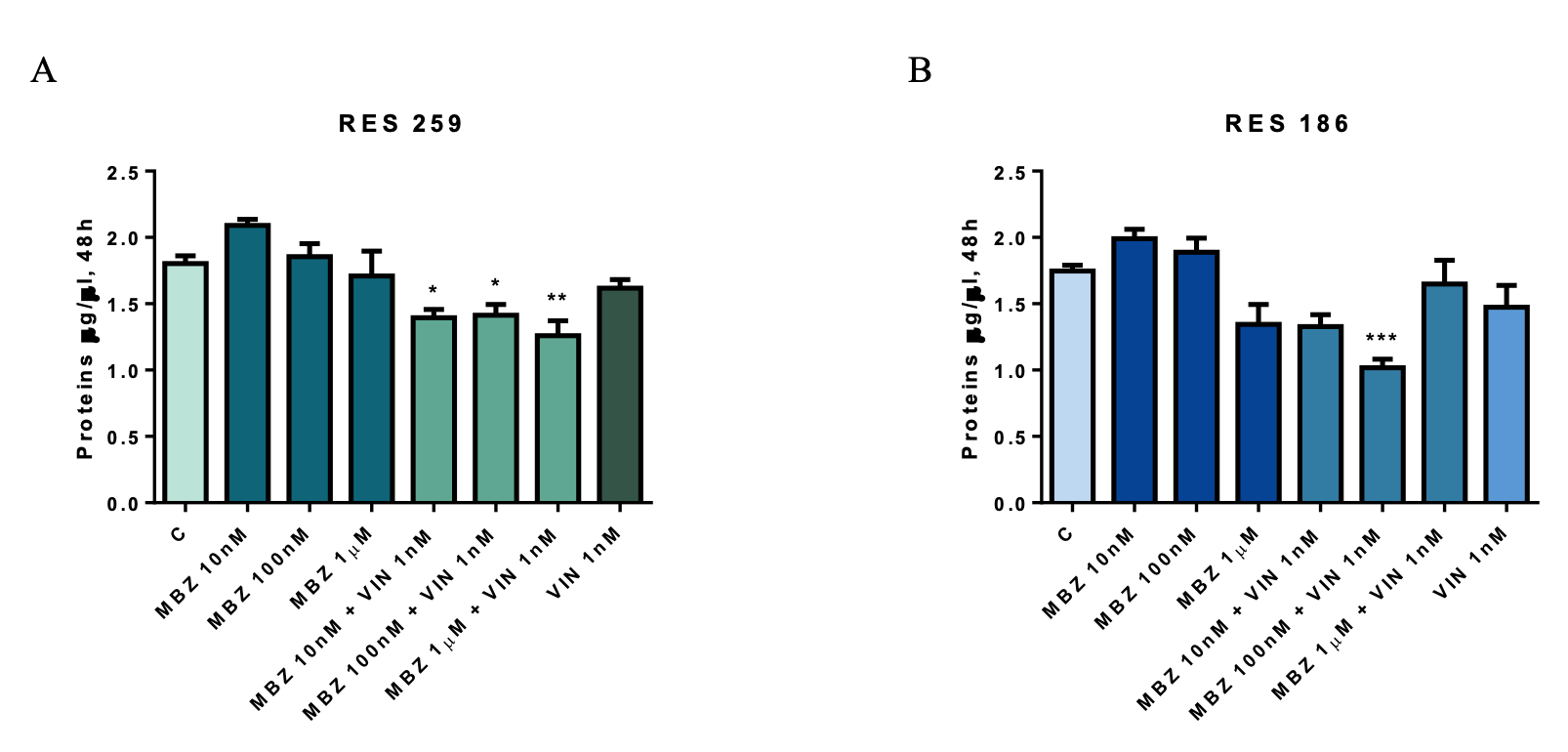
*

Supplementary Figure 5. Effect of Mebendazole on protein amounts (µg/µL) measured by the Bradford method after 48 h treatment in RES 259 and in RES 186. Protein amounts (µg/µL) were measured after 48 h of treatment by the Bradford assay and expressed relative to untreated cells (control). **(A)** RES 259 cells treated with: control, MBZ 100 nM, MBZ 1 µM, MBZ 100 nM + VIN 1 nM, MBZ 1 µM + VIN 1 nM, and VIN 1 nM. **(B)** RES 186 cells treated with the same conditions as in (A). Data are presented as mean ± SEM. Statistical analysis was performed using one-way ANOVA followed by Dunnett’s post hoc test. All p-values were calculated relative to the control sample. **p* < 0.05; ***p* < 0.01; ****p* < 0.001.


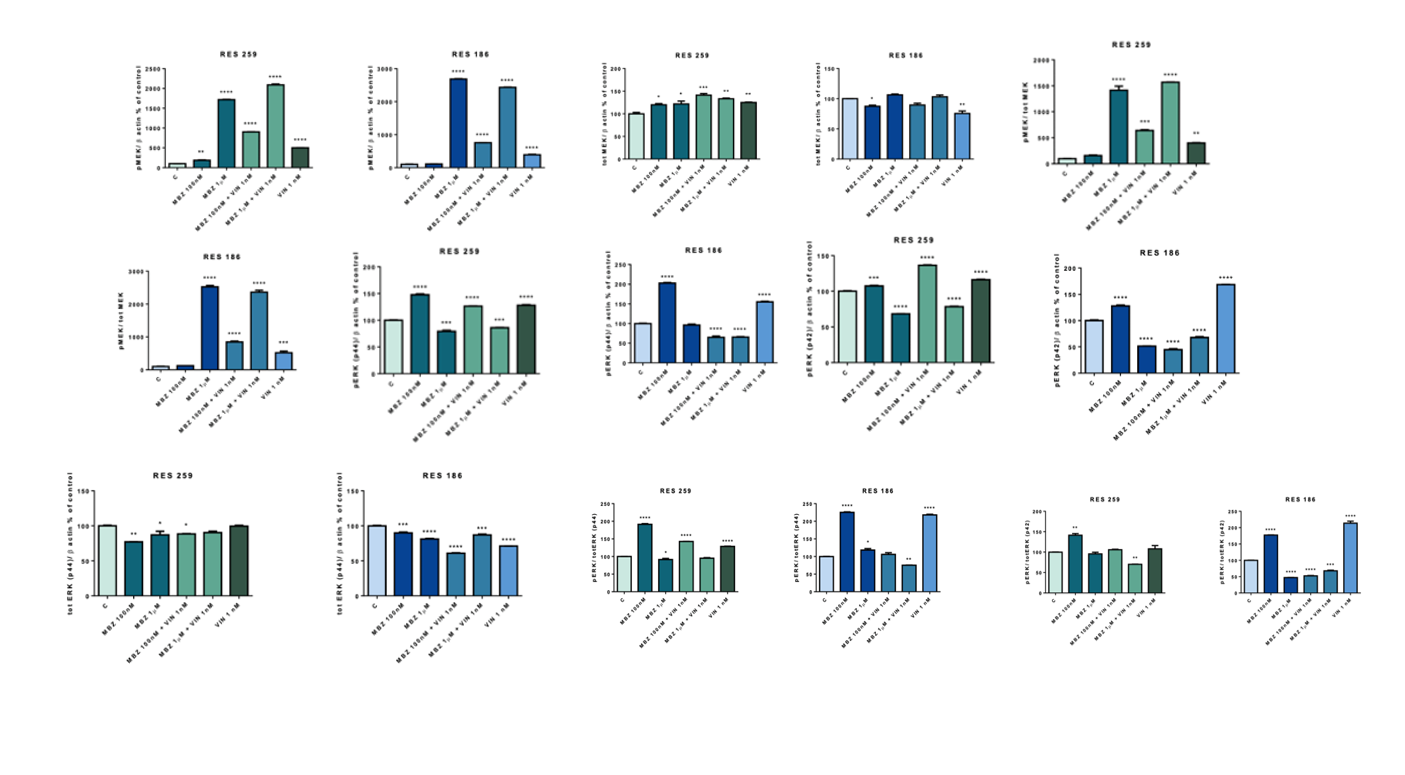


Supplementary Figure 6. Western blot analysis in RES 259 and RES 186 of some proteins involved in the MAPK pathway after 48 h of the following treatments: control, Mebendazole 100 nM, Mebendazole 1 μM, Mebendazole 100 nM and Vinblastine 1 nM, Mebendazole 1 μM and Vinblastine 1 nM, Vinblastine 1 nM. Values represent the mean ± standard error (SEM) and were evaluated by a one-way ANOVA followed by Dunnett's post-test. * *p* < 0.05; ** *p* < 0.01; *** *p* < 0.001; **** *p* < 0.0001. For every protein set, β-actin is reported as the normalizer gene.
